# Profound gastric mucosal changes and severe rebound acid hypersecretion after long‐term Vonoprazan use: A case report

**DOI:** 10.1002/deo2.70046

**Published:** 2024-12-21

**Authors:** Hiroko Suda, Sachi Eto, Koichi Sakurai

**Affiliations:** ^1^ Hattori Clinic Kumamoto Japan

**Keywords:** gastric mucosa, gastroesophageal reflux, proton pump inhibitors, rebound acid hypersecretion, Vonoprazan

## Abstract

Vonoprazan is a novel acid blocker with greater potency than proton pump inhibitors. A Japanese study reported no significant safety concerns over 5 years of Vonoprazan use; however, elevated serum gastrin and increased parietal cell and foveolar hyperplasia were observed, and long‐term safety data beyond 5 years are limited. We report a case that used Vonoprazan for 6 years, complicated by significant gastric epithelial changes during treatment and acute duodenal mucosal lesions following its discontinuation. A 76‐year‐old, treated with proton pump inhibitors for over 10 years, was switched to Vonoprazan due to his worsening symptoms. After its use, hemorrhagic hyperplastic polyps became prominent. Given concerns about Vonoprazan's effect on the gastric epithelium, the medication was changed to high‐dose H2 blocker therapy. Two months later, the patient complained of vomiting and black tarry stools. Esophagogastroduodenoscopy revealed a significant reduction of gastric polyps but multiple erosions and ulcers in the duodenum. This case indicates the potent effects of Vonoprazan on the gastric mucosa and the risk of severe rebound acid hypersecretion after its long‐term use.

## 1 INTRODUCTION

Vonoprazan, a potassium‐competitive acid blocker, inhibits H^+^/K^+^ ATPase with greater potency than proton pump inhibitors (PPIs). It offers several advantages over PPIs, including stability in acidic conditions, unaffected by food intake, quicker onset of acid suppression, and resistance to CYP2C19 polymorphism. A Japanese randomized controlled trial evaluating the safety of Vonoprazan over 5 years for gastroesophageal reflux disease (GERD) maintenance therapy, reported no increased risk of neoplastic changes and enterochromaffin‐like (ECL) hyperplasia, although elevated serum gastrin, and higher incidence of parietal cell and foveolar hyperplasia were noted compared to PPI.^1^

Endoscopic findings associated with long‐term PPI use, such as fundic gland polyps, hyperplastic polyps, multiple white and flat elevated lesions, cracked and cobblestone‐like mucosa, and black spots, are well‐known. Vonoprazan use has been reported to show additional unique features, including gastric mucosal redness, white spots, and web‐like mucus.^2^

Given the stronger acid inhibition and higher hypergastrinemia driven by Vonoprazan, its long‐term safety remains a concern. In this report, we present a case of a 76‐year‐old man who used Vonoprazan for 6 years, complicated by significant gastric epithelial changes, and acute duodenal mucosal lesions (ADML) that developed after switching from Vonoprazan to histamine type 2 receptor antagonist (H2‐blocker).

## 2 CASE REPORT

**FIGURE 1:**
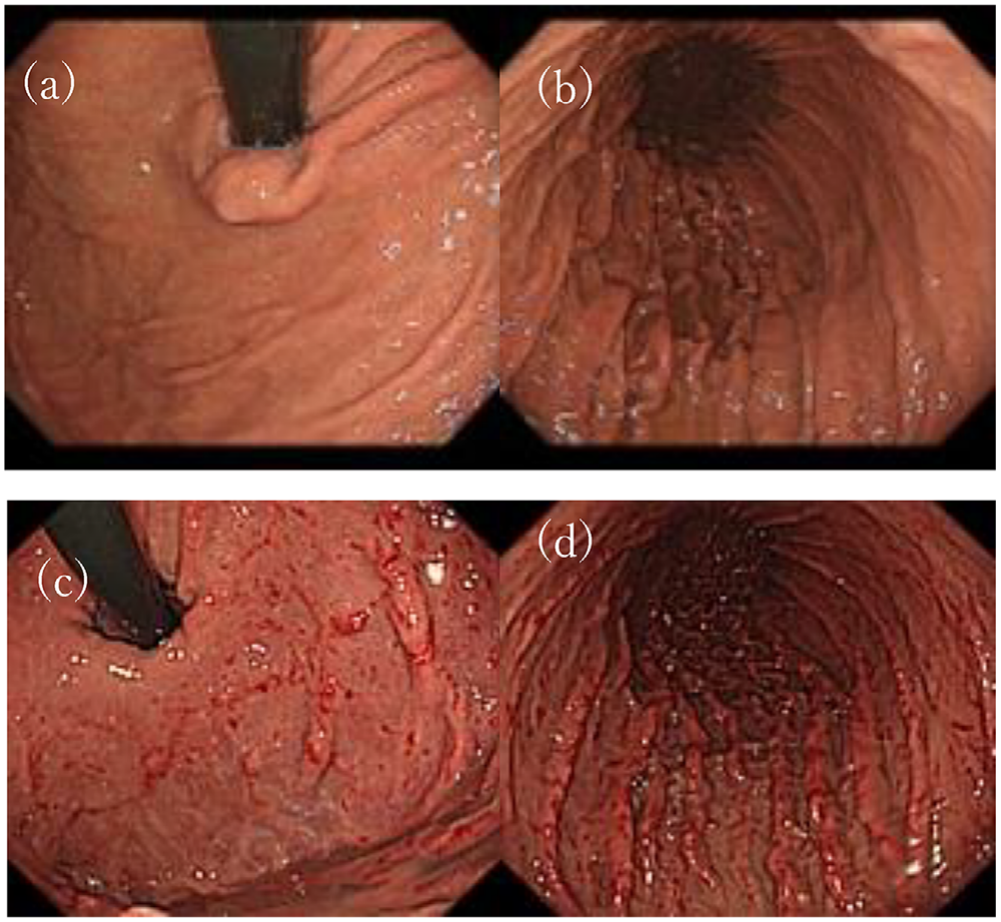
(a–d) Endoscopic images (a, b) before the initiation of Vonoprazan therapy. (c, d) after 19 months of Vonoprazan use, linear and spotty redness and multiple small hyperplastic polyps in the body became prominent in addition to previously observed fundic gland polyps.

**FIGURE 2:**
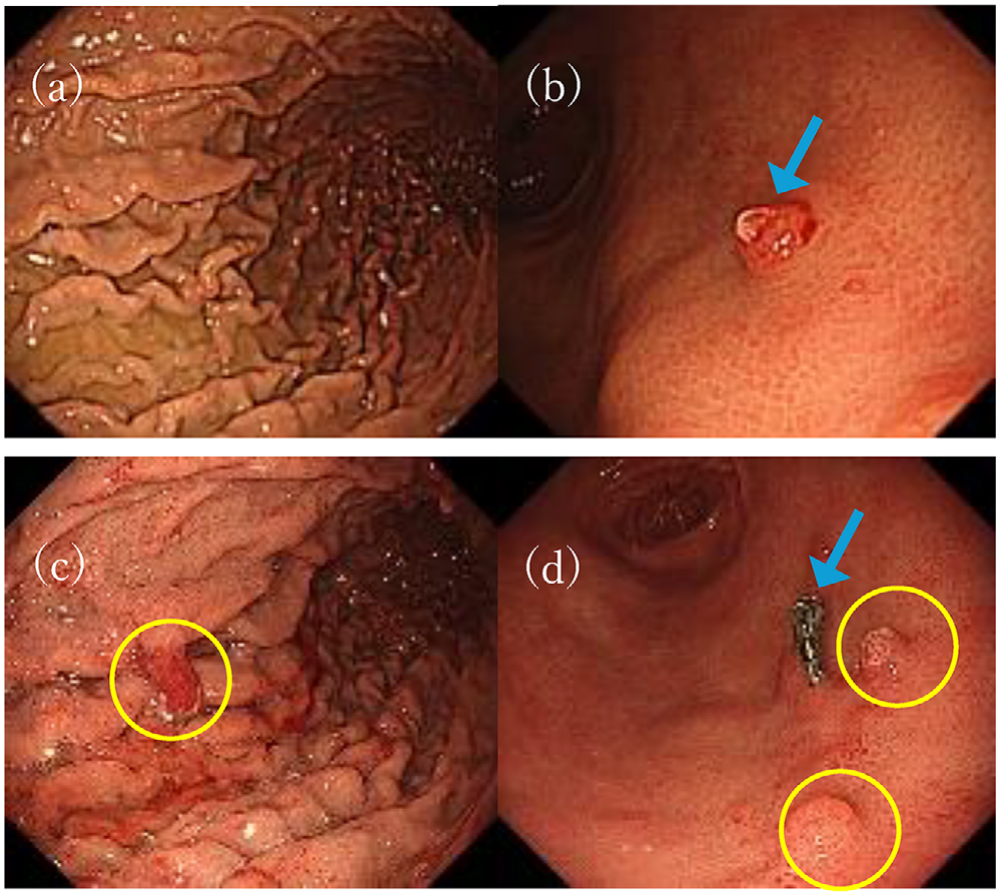
(a–d) Endoscopic images (a, b) after 59 months of Vonoprazan use, showing a hemorrhagic polyp (blue arrow) before resection. (c, d) After 69 months of Vonoprazan use, revealing the growth of new polyps was growing following the resection (yellow circle).

A 76‐year‐old man with a history of hypertension and post‐operation of thoracoabdominal aortic aneurysm had been receiving anti‐acid medication for heartburn for over 10 years. He has been prescribed antihypertensive and antilipidemic medications, without the use of anticoagulants or non‐steroidal anti‐inflammatory drugs. His liver and renal function were within normal limits. His *Helicobacter pylori* status was determined to be negative based on a negative serum anti‐*H. pylori* antibody test (< 3 U/mL) and a lack of mucosal atrophy on endoscopic examination. Initially treated with Omeprazole 20 mg, his medication was switched to Esomeprazole 20 mg 7 years ago due to worsening symptoms. Despite this change, Esomeprazole failed to adequately control his reflux symptom, even though endoscopic evaluation revealed grade N. Consequently, treatment with Vonoprazan 20 mg was initiated 6 years ago. His symptoms resolved soon after starting Vonoprazan; however, red hyperplastic polyps became prominent in addition to previously observed fundic gland polyps (Figure 1). After 59 months on Vonoprazan, a polypectomy was performed to remove hemorrhagic hyperplastic polyps. Despite the intervention, other hyperplastic polyps continued to grow (Figure 2). Suspecting a potential influence of Vonoprazan, the medication was switched to H2 blocker 20 mg twice daily, following a two‐week temporary transition period with Esomeprazole 20 mg (Supporting Information Figure).

One month later, the patient complained of mild heartburn and diarrhea. At the 2‐month follow‐up, his symptoms had worsened, with vomiting, black tarry stools, and a 7 kg weight loss. Physical examination revealed a slightly distended abdomen with tenderness from the epigastric region to the lower abdomen. Laboratory tests showed progressive anemia and elevated blood urea nitrogen levels, suggesting possible upper gastrointestinal bleeding (Supporting Information Table).

**FIGURE 3:**
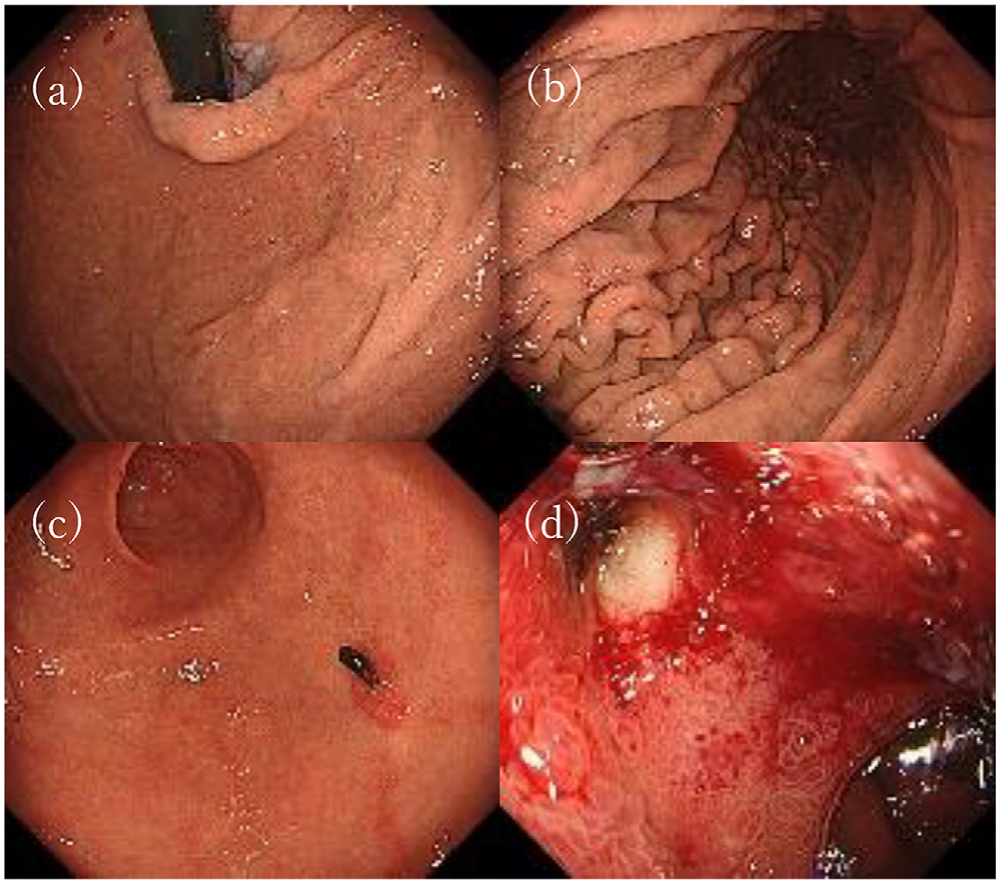
(a–c) Endoscopic images after switching to H2 blockers, showing a significant deduction of polyps in the stomach. (d) Endoscopic images revealing ulcers in the duodenum. This image shows an ulcer located in the superior duodenal angle.

Esophagogastroduodenoscopy revealed a significant reduction in the size and number of gastric polyps. However, multiple erosions, including 10 mm ulcers, were observed from the superior duodenal angle to the descending part of the duodenum (Figure 3). Additionally, the patient's reflux esophagitis, which had been grade N during Vonoprazan therapy, had worsened to grade B.

**FIGURE 4:**
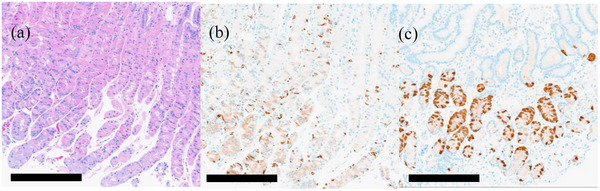
(a–c) Histological images. (a) Hematoxylin and eosin staining showing parietal cell hyperplasia in the gastric body, (b) Anti‐synaptophysin staining showing increased enterochromaffin‐like cells in the gastric body, and (c) Anti‐gastrin staining showing G cell hyperplasia in the antrum. Scale bars; 250 µm.

H2 blocker was switched back to Vonoprazan to manage the duodenal ulcers, leading to a rapid solution of symptoms. Subsequent endoscopy performed 1 month after restarting Vonoprazan confirmed improvement of ADML. Histological analysis of gastric tissue showed parietal cell hyperplasia in the corpus of the upper greater curvature and G‐cell hyperplasia in the antrum of the upper greater curvature (Figure 4). Following this confirmation, the patient's medication was changed to esomeprazole 20 mg daily. After approximately 3 months, there were no notable changes in symptoms or endoscopic findings.

During the clinical course, the patient's serum gastrin levels were measured as 481 pmol/L while on Vonoprazan, 100 pmol/L on Esomeprazole, and 80.6 pmol/L on an H2 blocker.

## 3 DISCUSSION

This case highlights significant gastric mucosal changes in a patient with 6 years of Vonoprazan use, coinciding with the severe hyperacid disorder during the de‐prescribing process of Vonoprazan. It offers insight into the potential complications of long‐term Vonoprazan therapy and its withdrawal.

Vonoprazan appears to have a more intense effect on the gastric mucosa compared to PPIs. In this case, the development of reddish hyperplastic polyps after switching from PPI to Vonoprazan, and their prompt regression within 2 months during transitioning to H2‐blocker, indicates that Vonoprazan has a more pronounced effect on the gastric mucosa than other acid‐suppressing drugs. While a few case reports have described a reduction in the number and size of gastric polyps about one year after discontinuation of Vonoprazan,^3^ the strikingly rapid mucosal changes observed in this case are notable. Furthermore, the positive correlation between elevated serum gastrin levels and the growth of hyperplastic polyps in this patient is consistent with a previous report showing that higher gastrin levels increased the risk of hyperplastic polyps in PPI users.^4^

This case demonstrates the risk of rebound acid hypersecretion (RAHS) after discontinuing long‐term Vonoprazan use. Despite high‐dose H2 blocker replacement therapy, the patient developed ADML. The acid‐inhibitory effect of the H2 blocker was insufficient to suppress his acid secretion. RAHS is characterized by acid hypersecretion status following the withdrawal of long‐term acid‐suppressive therapy, driven by the trophic effects of hypergastrinemia on ECL and parietal cells. Studies in mice have shown a relatively long lifetime of these cells; the lifetime of parietal cells exceeds 300 days,^5^ while ECL cells have a cell cycle time of approximately 60 days.^6^ Therefore, acid hypersecretion can persist until these cell populations gradually return to baseline levels, even after rapid normalization of elevated serum gastrin levels following discontinuation of acid‐suppressing drugs. Fossmark et al. showed that RAHS can last from 8 to 26 weeks after stopping PPI medication.^7^ In this case, the patient's prolonged use of Vonoprazan likely exacerbated the severity of RAHS. His histological findings, which demonstrated prominent parietal and G cell hyperplasia are compatible with this theory, though ECL cell hyperplasia was less pronounced in comparison. Another possible reason for the severity of his RAHS is his *H. pylori*‐negative status. Gillen et al. showed that RAHS occurred in *H. pylori*‐negative subjects after PPI treatment, while RAHS was partially masked in *H. pylori*‐positive subjects.^8^ Since *H. pylori* inhibits acid production through multiple mechanisms—such as direct damage to the gastric epithelial cells, stimulation of proinflammatory cytokines, suppression of H^+^/K^+^‐ATPaseαsubunit transcription, and enhancement of somatostatin release, it is plausible that acid rebound is more prominent in *H. pylori*‐negative patients.

Tanaka et al. reported that the discontinuation of Vonoprazan/PPI resulted in reflux symptoms in 28.3% of cases, with the severity and frequency of symptoms being higher in the Vonoprazan group compared to the PPI group, although the difference was not statistically significant.^9^ Our case supports these findings and is particularly noteworthy, as it demonstrates rapid gastric epithelial changes and the occurrence of a more severe complication, in addition to reflux symptoms.

Although the effect of long‐term PPI use before Vonoprazan cannot be overlooked, the potent effect of Vonoprazan likely exacerbated the acid hypersecretion in the gastric epithelium. While numerous studies have examined RAHS, the underlying risk factors remain incompletely understood.^10^ Based on the physiological mechanisms of RAHS, which is driven by hypergastrinemia induced by anti‐acid drug use, unnecessary high‐intensity or extended‐duration therapy should be avoided. Additionally, distinguishing between recurrence that warrants reinitiation of acid‐suppressive therapy and transient hyperacidity following withdrawal of these drugs can be challenging. Physicians should remain vigilant about the potential for RAHS, educate patients about this risk, and emphasize the importance of careful monitoring during and after deprescribing anti‐acid medications. For patients with a history of long‐term anti‐acid drug use, particular caution is necessary to safely taper or discontinue therapy, given the potential for severe complications like this case.

## CONFLICT OF INTEREST STATEMENT

None

## Supporting information



Laboratory data: 69month after utilizing Vonoprazan
