# Supplementary material for: Profound gastric mucosal changes and severe rebound acid hypersecretion after long‐term Vonoprazan use: A case report
Source: DEN Open. 2024 Dec 21;5(1):e70046. doi: 10.1002/deo2.70046 (PMC11662994; doi:10.1002/deo2.70046)
Supplement: Supplementary file 1 — Laboratory data: 69month after utilizing Vonoprazan [file DEO2-5-e70046-s001.docx]

Supplementary Figure


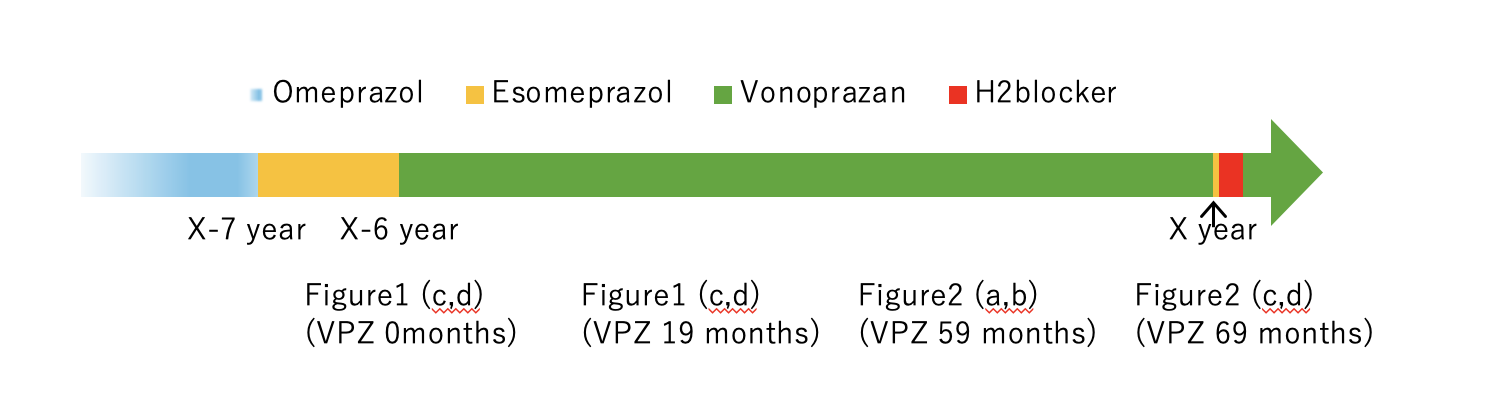


Supplementary Table

Laboratory data: 69month after utilizing Vonoprazan

| WBC | 6000 | /uL | TP | 6.5 | g/dL |
| --- | --- | --- | --- | --- | --- |
| RBC | 441 | ×10^4^/uL | Alb | 4.0 | g/dL |
| Hgb | 14.1 | g/dL |  |  |  |
| Hct | 39.7 | % | AST | 29 | IU/L |
| Plt | 22.2 | ×10^4^/uL | ALT | 21 | IU/L |
| MCV | 90 | fL | LDH | 242 | IU/L |
| MCH | 32.0 | pg | ALP | 79 | IU/L |
| MCHC | 35.5 | g/dL | γ-GTP | 104 | IU/L |
|  |  |  | T-Bil | 0.9 | g/dL |
| Na | 143 | mEq/L |  |  |  |
| K | 3.3 | mEq/L | BUN | 14.8 | mg/dL |
| Glu | 121 | mg/dL | Cre | 1.01 | mg/dL |

Laboratory data: 2 months after discontinuation Vonoprazan

| WBC | 10900 | /uL | TP | 6.3 | g/dL |
| --- | --- | --- | --- | --- | --- |
| RBC | 398 | ×10^4^/uL | Alb | 3.5 | g/dL |
| Hgb | 12.4 | g/dL |  |  |  |
| Hct | 37.6 | % | AST | 16 | IU/L |
| Plt | 27.3 | ×10^4^/uL | ALT | 16 | IU/L |
| MCV | 94 | fL | LDH | 183 | IU/L |
| MCH | 31.2 | pg | ALP | 78 | IU/L |
| MCHC | 33.0 | g/dL | γ-GTP | 36 | IU/L |
|  |  |  | T-Bil | 0.4 | g/dL |
| Na | 143 | mEq/L |  |  |  |
| K | 4.3 | mEq/L | BUN | 27.8 | mg/dL |
| Glu | 123 | mg/dL | Cre | 1.00 | mg/dL |
